# Current Challenges in Home Nutrition Services for Frail Older Adults in Japan—A Qualitative Research Study from the Point of View of Care Managers

**DOI:** 10.3390/healthcare1010053

**Published:** 2013-09-12

**Authors:** Yoshihisa Hirakawa, Takaya Kimata, Kazumasa Uemura

**Affiliations:** 1 Center for Postgraduate Clinical Training and Career Development, Nagoya University Hospital, 65 Tsuruma-cho, Showa-ku, Nagoya, Aichi 466-8560, Japan; E-Mail: kuemura@med.nagoya-u.ac.jp; 2 Aoi Home Clinic, 1-15 Hinokuchi-cho, Nishi-ku, Nagoya City, Aichi 451-0034, Japan; E-Mail: aoi.zaitaku@gmail.com

**Keywords:** nutrition, caregiver burden, home visit, frail older adults, care manager

## Abstract

Preventive care for frail older adults includes providing tailor-made diet information suited to their health conditions. The present study aims to explore the current situation and challenges of home nutrition advice for Japanese frail older adults using qualitative data from a ten-person group discussion among care managers. As the results of our analysis, nine themes were identified: (1) Homebound older adults develop poor eating habits; meals turn into a lonely and unpleasant experience; (2) With age, people’s eating and drinking patterns tend to deteriorate; (3) Many older adults and their family know little about food management according to condition and medication; (4) Many older adults do not understand the importance of maintaining a proper diet; (5) Many homebound older adults do not worry about oral hygiene and swallowing ability; (6) Some older adults are at high risk for food safety problems; (7) Only a limited range of boil-in-the-bag meal options are available for older adults; (8) Many older adults feel unduly confident in their own nutrition management skills; and (9) For many family caregivers, nutrition management is a burden. We conclude that the provision of tailor-made information by skilled dietitians and high-quality home-delivered meal service are essential for the successful nutrition management of the older adults.

## 1. Introduction

Due to the aging of the Japanese population, as well as the changing preferences of older patients and their families, a growing number of older people are now opting to spend the last years of their life in a community setting such as their own home [1]. In addition, the growth of the nuclear family in recent years has triggered an increase in the number of aged households, generating a progressive decline in the quality of the living environment of homebound older adults [2]. As a result, the community is expected to assume a growing responsibility in caring for frail older adults [1]. Thus, improving the quality and quantity of preventive care provision in the home has become an urgent priority in Japan.

Basic preventive care for the older adults includes providing nutritional advice and support, although this is often a complex and delicate task. For example, older patients with lifestyle-related diseases such as diabetes mellitus or hyperlipidemia need to have specific dietary requirement, while those with protein-energy malnutrition (PEM) need to follow an enhanced diet. Frail older adults are especially prone to PEM due to appetite loss and a decline in chewing power and digestive function, among other issues [1,3]. A number of studies have suggested that older adults with PEM are likely to perform less activities of daily life, thereby becoming more dependent on others [4,5,6]. Thus, frail older adults need tailor-made diet information specifically suited to their health conditions.

There are important challenges in the provision of information on eating habits and diet at home. In year 2000, Japan introduced a special care management system under its public long-term care insurance plan, granting care managers the responsibility of outlining long-term care plans for the older adults [7]. In 2009, we conducted a survey to find out about the kind of information family caregivers of homebound older patients normally seek and the way in which they generally obtain this information [8]. A total of 475 family caregivers of homebound older patients took part in the survey, and the results indicated that the respondents received health information either from their physician or from a care manager, despite the fact that the latter is not a recognized medical professional.

Under Japan’s public long-term care insurance system, homebound older adults are entitled to comprehensive nutritional support during home visits [9]. The program aims for a dietician to provide homebound older adults and their family with tailor-made nutrition services. Unfortunately, few older people actually benefit from the program because a large number of care managers are not aware of the advantages of nutritional guidance [9].

It is essential that home frail older adults and their family be provided with the appropriate nutritional guidance based on their specific needs [3,9]. As care service coordinators and health-related information providers, care managers can offer useful insight on the current situation and challenges of home nutrition advice for frail older adults.

There are unfortunately very few studies on this topic. The present study aims to explore the current situation and challenges of home nutrition service for Japanese frail older adults using qualitative data from a focus group discussion among care managers.

## 2. Method

### 2.1. Study Participants

We randomly recruited 10 non-nurse care managers from different home care support centers related to Nagoya University Hospital, considering a wide range of characteristics such as age and workplace among the participants (Table 1). The Japanese long-term care system provides care managers with official recognition as professionals whose primary responsibility is to oversee the coordination of care services and the formulation of care plans for older people who require care prevention services or nursing care. Licensed professionals such as nurses, social workers, professional caregivers, can be certified as “care managers” provided they go through a special training program [1].

**Table 1:** Participants’ characteristics.

Participant	Age (year)	Sex (F/M)	Career (year)
1	54	F	4
2	50	F	7
3	48	F	2
4	45	F	7
5	44	F	4
6	43	M	3
7	36	M	5
8	34	F	2
9	32	M	6
10	29	F	0.4

### 2.2. Data Collection

We collected qualitative data through ten-person discussions in March 2013. First, the 10 care managers took part in a 60-minute discussion on the eating habits of home frail older adults, jotting down all of their initial ideas and thoughts on the topic. Second, based upon these written notes, the participants spent another 30-minute session further exchanging on the issue until the discussion no longer yielded new ideas or thoughts. Third, based upon these written notes, each participant drew up a complete list of all the ideas and thoughts generated during the discussions. Finally, the participants’ ideas and thoughts were transferred onto 154 labels (*i.e.*, a short phrase or sentence to summarize each unique idea or thought).

### 2.3. Analysis Using the KJ Method

We used the KJ method as a qualitative research tool. The concept and background of the method was explained elsewhere [10,11]. In summary, the KJ method (Kawakita’s initials) was created in the 1960s by Japanese ethnologist Jiro Kawakita. The KJ method is now widely employed in Japan as a tool for qualitative research and improvement of business operation. The KJ method allows for information and ideas to be synthesized into a conceptual visual map using labels [10,11,12].

**Figure 1:**
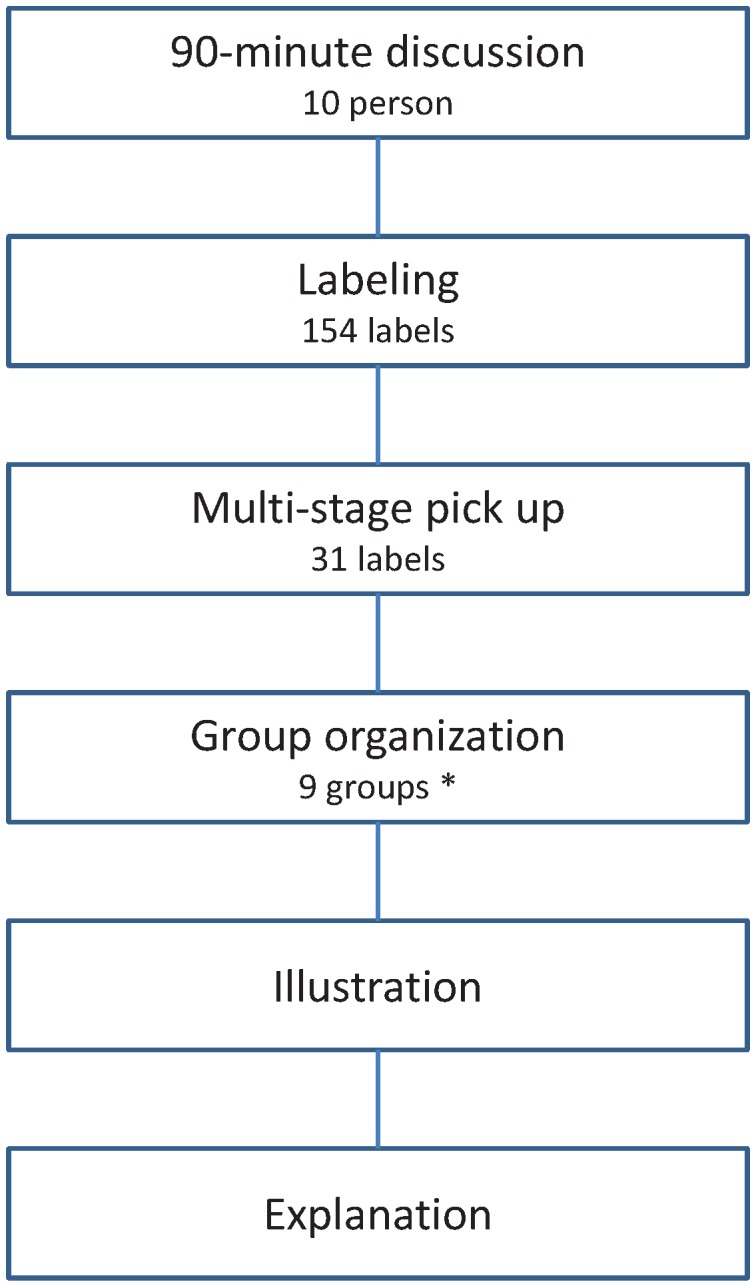
Procedure of KJ method.

The analysis (called “abduction” in the KJ method) was performed in March 2013 by the first author. As mentioned above, we compiled a total of 154 labels reflecting the thoughts and experiences of care managers on home end-of-life care provision (Figure 1). The KJ method makes it possible to quickly and efficiently frame key concepts into labels. The KJ method allows specially trained practitioners to select the number of labels carefully while saving time and labor. This procedure is called the multi-stage pick-up procedure. First, the author picked up 31 out of 154 labels, using the following procedure: (1) decide on a target number of labels; (2) read the labels silently and memorize them to get an overall impression; (3) mark the labels we wish to keep; (4) in a second round of pick-up, mark the labels we wish to keep among the previously selected labels; (5) repeat this process until the resulting number of labels is close to the target number; and (6) carefully select final labels. The target number of labels for the multi-stage pick-up procedure is generally required to be more than one-fifth of all labels to ensure the quality of the study. Second, the author organized the remaining 31 labels into groups using the following KJ method procedure: (1) read the labels silently to grasp the entire image; (2) combine labels that share a strong similarity in substance; (3) set aside any label that stands apart (“loner”); (4) make a “first-step nameplate” for each group of labels (*i.e.*, a short phrase or sentence to summarize the theme of the labels); (5) again, read the loner labels and first-step nameplate labels silently and combine labels that share a strong similarity in substance; (6) set aside any labels that stand apart; and (7) after further reflection, make a “second-step nameplate” for each group of labels. When grouping the labels, we endeavored not to follow standardized, stereotypical perceptions. Because the KJ method treats loner labels as separate groups, we ended up with nine groups (including three loner labels) following group organization. Third, we arranged the groups with nameplates and the loner labels onto a large sheet of paper, paying close attention to the inter-relationships between each group. Finally, to confirm the clarity and cohesion of the group arrangements to study participants, we added a title that captured the overall message of the illustration and the relationships among the organized groups and loner labels.

* including 3 loner labels.

## 3. Results

**Figure 2:**
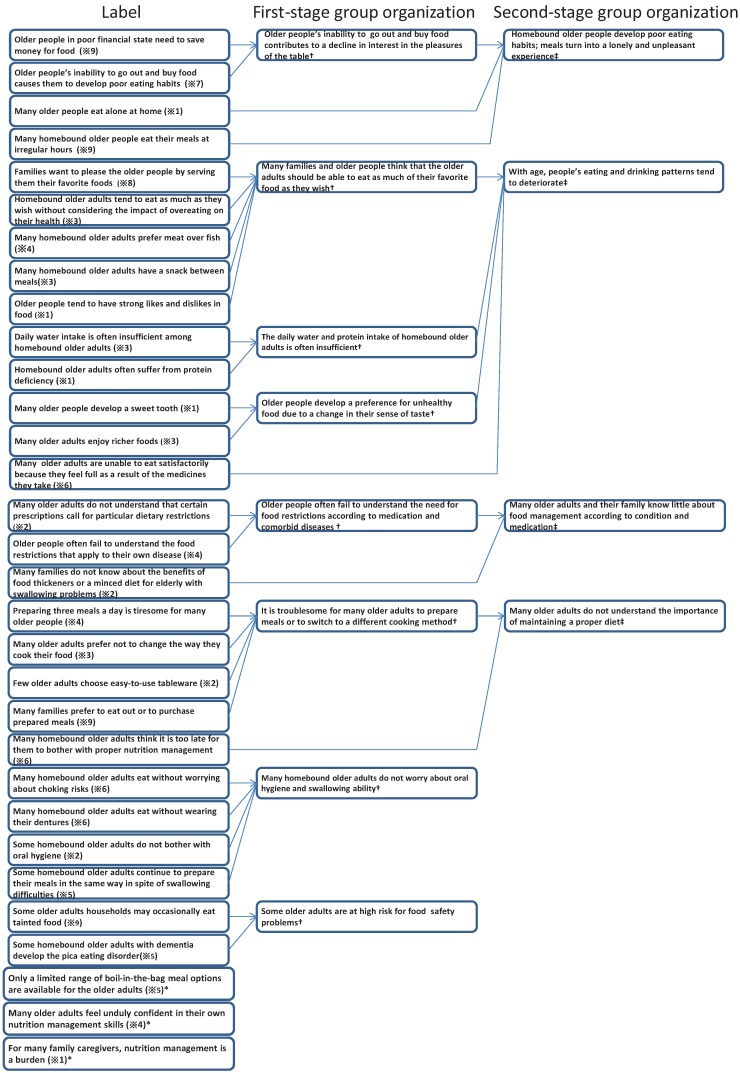
Flowchart of group organization.

As shown in Figure 2, the author organized the 31 labels into groups using the KJ method procedure, and nine groups (including three loner labels) were extracted. Our results suggest that a wide range of nutrition management issues among older adults increases the caregiver’s burden.

※1–10: participant number, *: loner label, †: 1st-step nameplate, ‡: 2nd-step nameplate.

### 3.1. Group Organization

(1) Homebound older adults develop poor eating habits; meals turn into a lonely and unpleasant experience.

The first-step nameplate “Older people’s inability to go out and buy food contributes to a decline in interest in the pleasures of the table”, and the labels “Many older people eat alone at home” (Participant and “Many homebound older people eat their meals at irregular hours” (Participant 9) suggest that homebound older adults develop poor eating habits.

(2) With age, people’s eating and drinking patterns tend to deteriorate.

This group includes the label “Many older adults are unable to eat satisfactorily because they feel full as a result of the medicines they take” (Participant 6) and the following first-step name plates: “Many families and older people think that the older adults should be able to eat as much of their favorite food as they wish”, “The daily water and protein intake of homebound older adults is often insufficient”, and “Older people develop a preference for unhealthy food due to a change in their sense of taste”.

(3) Many older adults and their family know little about food management according to condition and medication.

This group includes three labels: “Many older adults do not understand that certain prescriptions call for particular dietary restrictions” (Participant 2), “Older people often fail to understand the food restrictions that apply to their own disease” (Participant 4), and “Many families do not know about the benefits of food thickeners or a minced diet for older adults with swallowing problems” (Participant 2).

(4) Many older adults do not understand the importance of maintaining a proper diet.

The labels “Preparing three meals a day is tiresome for many older people” (Participant 4), “Many older adults prefer not to change the way they cook their food” (Participant 3), “Few older adults choose easy-to-use tableware” (Participant 2), and “Many families prefer to eat out or to purchase prepared meals” (Participant 9) were placed into a first-step group with the nameplate “It is troublesome for many older adults to prepare meals or to switch to a different cooking method”. This group also includes the label “Many homebound older adults think it is too late for them to bother with proper nutrition management” (Participant 6).

(5) Many homebound older adults do not worry about oral hygiene and swallowing ability.

First-step nameplates include: “Many homebound older adults eat without worrying about choking risks” (Participant 6), “Many homebound older adults eat without wearing their dentures” (Participant 6), “Some homebound older adults do not bother with oral hygiene” (Participant 2), and “Some homebound older adults continue to prepare their meals in the same way in spite of swallowing difficulties” (Participant 5). These nameplates suggest that many homebound older adults have little interest in maintaining good swallowing ability and keeping good oral hygiene.

(6) Some older adults are at high risk for food safety problems.

The labels “Some older adults households may occasionally eat tainted food” (Participant 9) and “Some homebound older adults with dementia develop the pica eating disorder” (Participant 5), which were placed into a separate group, emphasize the risk of pica among home frail or older adults with dementia. Pica is an eating disorder typically defined as the persistent ingestion of nonnutritive substances, and is commonly seen in demented patients.

(7) Loner labels

The labels “Only a limited range of boil-in-the-bag meal options are available for the older adults” (Participant 5), “Many older adults feel unduly confident in their own nutrition management skills” (Participant 4), and “For many family caregivers, nutrition management is a burden” (Participant 1) do not fall under any group.

### 3.2. Illustrated Figure

**Figure 3:**
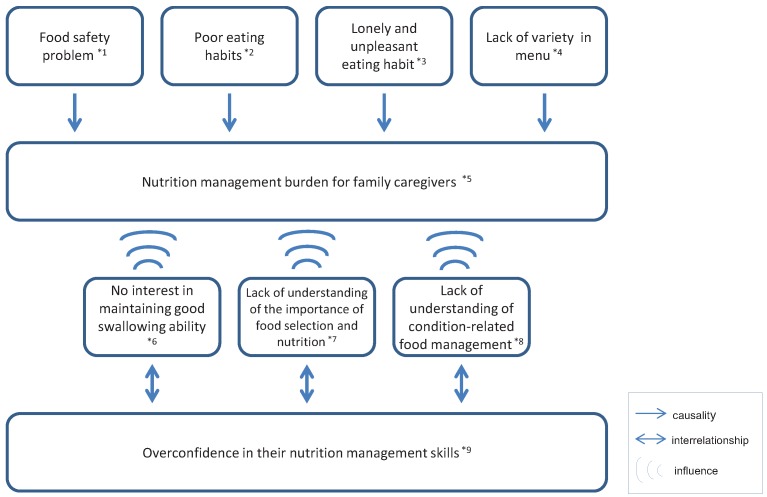
Current situation and challenges of home nutrition service for Japanese frail older adults: A wide range of nutrition management issues among older adults increases caregivers’ burden.

Our illustrated figure entitled “A wide range of nutrition management issues among older adults increases caregivers’ burden” suggests that a wide range of nutrition management issues including food safety, eating habits, mealtime social environment, variety of foods available, nutrition knowledge, and overconfidence contribute to family caregiver burden (Figure 3).

*1 Food safety problem: The catchphrase of 1st-step nameplate “Some older adults are at high risk for food safety problems”. *2 Poor eating habits: The catchphrase of 2nd-step nameplate “With age, people’s eating and drinking patterns tend to deteriorate”. *3 Lonely and unpleasant eating habit: The catchphrase of 2nd-step nameplate “Homebound older adults develop poor eating habits; meals turn into a lonely and unpleasant experience”. *4 Lack of variety in menu: The catchphrase of a label “Only a limited range of boil-in-the-bag meal options are available for the older adults”. *5 Nutrition management burden for family caregivers: The catchphrase of a label “For many family caregivers, nutrition management is a burden”. *6 No interest in maintaining good swallowing ability: The catchphrase of 1st-step nameplate “Many homebound older adults do not worry about oral hygiene and swallowing ability”. *7 Lack of understanding of the importance of food selection and nutrition: The catchphrase of 2nd-step nameplate “Many older adults do not understand the importance of maintaining a proper diet”. *8 Lack of understanding of condition-related food management: The catchphrase of 2nd-step nameplate “Many older adults and their family know little about food management according to condition and medication”. *9 Overconfidence in their nutrition management skills: The catchphrase of a label “Many older adults feel unduly confident in their own nutrition management skills”.

## 4. Discussion

We found that family caregivers feel a heavy burden of responsibility with regards to the nutrition management of the older adults under their care. This burden is even greater when the older adults suffer from dementia or have experienced a stroke [13,14,15]. As our results suggest, frail older adults people are often unable to go out and buy food and they tend to adopt poor eating habits and mealtime patterns. Thus, for the older adults, an adequate diet depends on the ability to procure and prepare food and to eat independently or on the availability of dietary assistance when needed. In addition, we found that the risk of pica possibly increases caregiver burden. Pica is fairly common in older adults with dementia although this trend has not yet been systematically studied [14]. Changes in eating habits are of clinical importance since they can seriously affect the patients’ physical health and are a major source of concern for caregivers [16]. Nutrition education may help reduce caregiver stress and maintain the caregivers’ health and well-being [17]. Family caregivers of frail older adults who require care should be given counseling and education on nutrition management for frail older adults. 

Moreover, we found that caregiver burden was also affected by the limited availability of boil-in-the-bag meal options. While boil-in-the-bag meals offer a suitable alternative to regular meals cooked from fresh ingredients and help reduce caregiver stress, our previous study suggests that a number of family caregivers prefer not to resort to boil-in-the-bag or frozen meals because they find them unhealthy or undesirable [18]. However, we believe that ready-to-eat meals are a practical solution that helps lessen the caregiver’s burden, and that family caregivers should be educated on how to choose and use them. We also believe that home-delivered meals service can make a significant contribution to the nutrient intakes of the homebound, providing meals to housebound consumers. Although the effectiveness of such service is not well confirmed, some studies suggested that using the home-delivered meal service may create recipient satisfaction [19,20]. Such service will help lessen the caregiver’s burden of preparing a meal for their older adults.

Our study also indicates that some homebound older adults are overly confident in their ability to manage their diet and do not recognize the necessity of any kind of nutritional advice. Although good food choices and a balanced diet are essential for older adults to maintain a healthy lifestyle, there are various obstacles that prevent older adults from practicing good eating habits, including loneliness, economic concerns, lack of cooking skills or desire to cook, inadequate nutritional knowledge, oral problems. For example, some studies suggested that older adults’ diets are low in calories and other nutrients [20]. In addition, Nam *et al.* [21] summarized existing knowledge regarding various barriers of diabetes mellitus management from the perspectives of both patients and clinicians in their review article, and suggested that patients’ adherence, attitude, beliefs, and knowledge about diabetes mellitus affect self-management. These findings suggest the need for tailor-made nutrition management according to the older adults’ living environment and physical assessment, with particular attention to the relation between older adults and family.

Under Japan’s public long-term care insurance system, home visit nutrition guidance by a dietitian is provided; namely, the older client and the family are entitled to receive a personalized education program on cooking and eating, in accordance with the visiting dietician’s assessment of the older adults’ condition and living environment [9,18]. Although this nutrition support service is certainly appropriate and much needed for homebound older adults and family to maintain healthy eating habits, the initiative is still insufficient in terms of quantity and quality [9]. We believe that the importance of home-based nutritional guidance by a dietician should be brought to the attention of a greater number of professionals including physicians, nurses, care managers, as well of as older clients and family members. Heightened awareness and involvement on the part of the medical community will lead to the implementation of public policies to increase the education and number of dietitians. We also think that the home-delivered meal service help older adults maintain a healthy lifestyle and independence. The meals were particularly helpful for those with special dietary requirements, such as diabetes [22].

There are several study limitations. First, the study participants were care managers from home care support centers related to Nagoya University Hospital. This study reflects the thoughts of care managers who are within a very limited area. Because the way people think varies according to where they live, we believe that additional studies are needed to determine whether our results also apply to other areas. We also think that the view or thought of the older adults and their families are valuable to us for understanding current challenges in home nutrition services for frail older adults. We should conduct the studies analyzing such samples. Lastly, the number of the participants was limited. In qualitative methods including the KJ method, study sample size is not very important. However, we may not have collected enough qualitative data from the 10 participants in this study. Additional qualitative studies should be conducted, and our results should be generalized with caution.

## 5. Conclusions

The present qualitative study reveals that a wide range of nutrition management issues including food safety, eating habits, mealtime social environment, variety of foods available, nutrition knowledge, and overconfidence increase family caregiver burden. For the nutrition management of the older adults to be successful, the provision of tailor-made and home-based information by skilled dietitians and high-quality home-delivered meal service are essential.

A wide range of nutrition management issues including food safety, eating habits, mealtime social environment, variety of foods available, nutrition knowledge, and overconfidence increase family caregiver burden.

## References

[1] Hirakawa Y., Esther C., Amanda J. (2012). Palliative care for the elderly: A Japanese perspective. Contemporary and Innovative Practice in Palliative Care.

[2] Kimura Y., Wada T., Okumiya K., Ishimoto Y., Fukutomi E., Kasahara Y., Chen W., Sakamoto R., Fujisawa M., Otsuka K. (2012). Eating alone among community-dwelling Japanese elderly: Association with depression and food diversity. J. Nutr. Health Aging.

[3] Endevelt R., Werner P., Stone O. (2006). Dietitians’ attitudes regarding elderly nutritional factors. J. Nutr. Elder..

[4] Soini H., Routasalo P., Lagström H. (2004). Characteristics of the Mini-Nutritional Assessment in elderly home-care patients. Eur. J. Clin. Nutr..

[5] Sharkey J.R. (2002). The interrelationship of nutritional risk factors, indicators of nutritional risk, and severity of disability among home-delivered meal participants. Gerontologist.

[6] Shatenstein B., Kergoat M.J., Reid I., Chicoine M.E. (2013). Dietary intervention in older adults with early-stage Alzheimer dementia: Early lessons learned. J. Nutr. Health Aging.

[7] Matsuda S., Yamamoto M. (2001). Long-term care insurance and integrated care for the aged in Japan. Int. J. Integr. Care.

[8] Hirakawa Y., Kuzuya M., Enoki H., Uemura K. (2011). Information needs and sources of family caregivers of home elderly patients. Arch. Gerontol. Geriat..

[9] Hirakawa Y., Masuda Y., Uemura K., Naito M., Kuzuya M., Iguchi A. (2003). Dietitians’ understanding of personalized nutritional guidance—Proposals to increase home visits by dietitians. Nippon Ronen Igakkai Zasshi.

[10] Mushinkan. http://mushin-kan.jp/.

[11] Raymond S. (1997). The KJ method: A technique for analyzing data derived from Japanese ethnology. Hum. Organ..

[12] Kawakita J. (1967). Abduction(Hassoho).

[13] Silva P., Kergoat M.J., Shatenstein B. (2008). Challenges in managing the diet of older adults with early-stage Alzheimer dementia: A caregiver perspective. J. Nutr. Health Aging.

[14] Sugiura K., Ito M., Mikami H. (2007). Family caregiver burden caused by behavioral and psychological symptoms of dementia: Measurement with a new original scale. Nippon Ronen Igakkai Zasshi.

[15] Hafsteinsdóttir T.B., Vergunst M., Lindeman E., Schuurmans M. (2011). Educational needs of patients with a stroke and their caregivers: A systematic review of the literature. Patient Educ. Couns..

[16] Fairburn C.G., Hope R.A. (1988). Changes in eating in dementia. Neurobiol. Aging.

[17] Silver H.J., Wellman N.S. (2002). Nutrition education may reduce burden in family caregivers of older adults. J. Nutr. Educ. Behav..

[18] Hirakawa Y., Enoki H., Uemura K. (2010). Proposal concerning personalized nutritional guidance. Nippon Ronen Igakkai Zasshi.

[19] Krassie J., Smart C., Roberts D.C. (2000). A review of the nutritional needs of Meals on Wheels consumers and factors associated with the provision of an effective meals on wheels service-an Australian perspective. Eur. J. Clin. Nutr..

[20] Joung H.W., Kim H.S., Yuan J.J., Huffman L. (2011). Service quality, satisfaction, and behavioral intention in home delivered meals program. Nutr. Res. Pract..

[21] Nam S., Chesla C., Stotts N.A., Kroon L., Janson S.L. (2011). Barriers to diabetes management: Patient and provider factors. Diabetes Res. Clin. Pract..

[22] Wellman N.S., Rosenzweig L.Y., Lloyd J.L. (2002). Thirty years of the older Americans Nutrition Program. J. Am. Diet. Assoc..

